# The Functionality of Spontaneous Mimicry and Its Influences on Affiliation: An Implicit Socialization Account

**DOI:** 10.3389/fpsyg.2016.00458

**Published:** 2016-03-31

**Authors:** Liam C. Kavanagh, Piotr Winkielman

**Affiliations:** ^1^Department of Psychology, University of California, San Diego, San DiegoCA, USA; ^2^Psychology, SWPS University of Social Sciences and HumanitiesWarsaw, Poland; ^3^Behavioural Science Group, Warwick Business School, University of WarwickCoventry, UK

**Keywords:** mimicry, imitation, contagion, social learning, implicit knowledge, socialization, non-verbal behavior

## Abstract

There is a broad theoretical and empirical interest in spontaneous mimicry, or the automatic reproduction of a model’s behavior. Evidence shows that people mimic models they like, and that mimicry enhances liking for the mimic. Yet, there is no satisfactory account of this phenomenon, especially in terms of its functional significance. While affiliation is often cited as the driver of mimicry, we argue that mimicry is primarily driven by a learning process that helps to produce the appropriate bodily and emotional responses to relevant social situations. Because the learning process and the resulting knowledge is *implicit*, it cannot easily be rejected, criticized, revised, and employed by the learner in a deliberative or deceptive manner. We argue that these characteristics will lead individuals to preferentially mimic ingroup members, whose implicit information is worth incorporating. Conversely, mimicry of the wrong person is costly because individuals will internalize “bad habits,” including emotional reactions and mannerisms indicating wrong group membership. This pattern of mimicry, in turn, means that observed mimicry is an honest signal of group affiliation. We propose that the preferences of models for the mimic stems from this true signal value. Further, just like facial expressions, mimicry communicates a genuine disposition when it is truly spontaneous. Consequently, perceivers are attuned to relevant cues such as appropriate timing, fidelity, and selectivity. Our account, while assuming no previously unknown biological endowments, also explains greater mimicry of powerful people, and why affiliation can be signaled by mimicry of seemingly inconsequential behaviors.

## Introduction

Science knows many facts about spontaneous mimicry – the unintentional reproduction of model’s behaviors, including gestures, postures, facial expressions, accents, and mannerisms. Yet, a functional explanation of this phenomenon is still elusive. Here we argue that, just like other forms of imitation, spontaneous mimicry primarily serves to acquire appropriate actions and reactions, including emotional responses, but in an implicit fashion (see Kavanagh, 2015 for initial formulation). We argue that several puzzling features of spontaneous mimicry derive from this learning function, including mimicy’s unconscious nature, its moderation by group membership and liking, models’ positive response to mimicry, mimicry’s low-fidelity, and its seemingly strategic use. Before discussing our account, we briefly review core empirical findings and theories.

## Human Mimicry: Six Core Empirical Findings and Some Distinctions

Research has discovered several features of mimicry (Chartrand and Lakin, 2013). First, it is *implicitly* produced and perceived. That is, individuals do not notice when they mimic others, or when others mimic them, and have limited conscious control, even with economic incentives (Belot et al., 2013). Second, *individuals like those who mimic them* (Chartrand and Bargh, 1999). Third, *individuals mimic people they know and like*, as when subjects imitate friends more than strangers (e.g., McIntosh, 2006). Fourth, *individuals mimic outgroup members less or even do the opposite*. For example, in competitive situations participants scowl to competitors’ smiles, just as quickly as they smile to teammates’ smiles (Lanzetta and Englis, 1989; Carr et al., 2014).

There are also puzzling aspects of spontaneous mimicry. One is that despite its implicit nature, it can be seemingly *strategic.* For example, an earlier failure to affiliate with one person spurs participants toward increased mimicry in a subsequent interaction with another person, especially when participants had an initial goal to affiliate (Lakin and Chartrand, 2003; see also Thelen et al., 1980; Wang and Hamilton, 2012). Additionally, mimicry is *subtle, approximate (low-fidelity)*, and *timing-dependent*. For example, *blatant, exact (high-fidelity), or ill-timed mimicry* typically backfires (e.g., Parrill and Kimbara, 2006; Ashton-James et al., 2007). Finally, mimicry can be triggered by relatively simple features of gestures and expressions, and thus elicited even by artificial agents (Bailenson and Yee, 2005; Hofree et al., 2014).

Some distinctions are important here. People spontaneously mimic movements, gestures, postures, and affective expressions. Clearly, forms of imitation differ. For example, rapid mimicry of finger movements involves simple matching of overtly visible actions, whereas mimicry of emotional expressions may also involve matching of correlated underlying states. However, our short piece will treat these phenomena as similar as they could all fulfill the functional role we assign to mimicry. Still, we recognize that our claims may be most applicable to affective mimicry, just because affect and emotions are most reflecting of preferences and values. Finally, it is worth remembering that not all implicit coordination phenomena involve mimicry and thus may require separate analysis.

## Extant Accounts of Spontaneous Mimicry: What is the Primary Function?

Several functional accounts of spontaneous mimicry have been proposed. All offer useful insights into possible functions, but struggle to accommodate some of the empirical facts discussed earlier. Here we mention a few accounts, though our abbreviated presentation does not capture their complexity. Our goal here is highlight the need for another type of functional explanation, which we propose shortly.

One class of accounts emphasizes mimicry’s function for learning, coordination and synchrony, underpinned by ideomotor mechanisms linking perception and action (e.g., Chartrand and Bargh, 1999; Preston and de Waal, 2002; Heyes, 2011). These accounts are elegant, yet they do not easily accommodate findings showing modulation by liking, strategic mimicry, and anti-mimicry of outgroup members. Lakin et al. (2003) agree that mimicry was originally an evolved learning mechanism, but later came to be a sort of “social glue” because it demonstrated similarity to others. This view of mimicry as an outward demonstration of “alikeness” is shared by Over and Carpenter (2012), who hold that the ability for mimicry to gain the favor of others is due to a biological disposition toward liking others who are like us. Supporting this view is evidence, for example, that participants are more attracted to others whose experimental code numbers share digits with their own birthdays (Jones et al., 2004). Note, however, that many cases of spontaneous mimicry cannot be explained as attempts to elicit the impression of similarity. For example, less powerful individuals mimic the more powerful individuals, but such mimicry actually signals differences in power, rather than similarity (Over and Carpenter, 2015). Also, as mentioned, mimicry tends to be imprecise, but the similarity account would suggest higher effectiveness of high-fidelity mimicry.

Wang and Hamilton (2012) posit a sophisticated process in their theory of Machiavellian mimicry. It suggests that people strategically exploit mimicry to achieve their affiliation goals and advance social standing with a model. This theory has trouble with unconscious aspects of mimicry. More problematically, it implies asymmetric levels of rationality between the mimic (who is smart) and the model (who is fooled) in an extended Machiavellian arms race. That is, if mimicry is Machiavellian, then why should models respond positively to it in the long run? The same concern applies to the idea that mimicry exploits an innate attraction to similar others. If mimicry only indicates surface similarity, then the long-term wisdom of affiliating with mimics is questionable.

## The Current Proposal

The current proposal, like the above accounts, assumes that mimicry solves a problem, and that there are mechanistic constraints on possible solutions. However, unlike previous accounts, we emphasize the functionality (i.e., ecological rationality) of *both* mimicry *and* model’s affiliation with mimics – that is, we seek to satisfy criteria of “symmetric functionality” of mimicry and affiliative responses to it. Further, we aim to make the account as parsimonious as possible. In fact, we do not posit any special skills beyond the capacity to link perception and action and modify these links based on environmental reward structures (Heyes, 2011). We believe that the problem that spontaneous mimicry primarily solves is, like other forms of imitation, a need to learn group- and environment-specific skills and actions – a problem particularly pronounced for humans living in separated, competitive groups. However, in this case the acquired knowledge is implicit and under limited conscious control. We further propose that it is rational to honestly and spontaneously internalize the implicit, non-causal knowledge of people who face similar environmental action-response contingencies. We also note that these criteria are approximated by ingroup membership. Further, we propose that mimicry is an ecologically valid signal of desire for affiliation. Strategic use of mimicry, we propose, follows straightforwardly from these assumptions. We elaborate on these points next.

## Spontaneous Mimicry is for Learning

Intentional imitation is considered an important tool for cultural learning, including learning of instrumental actions (Tomasello et al., 1993; Gergely and Csibra, 2005; Whiten et al., 2009). However, note that similar imitative learning mechanism are also involved in acquisition of implicit *bodily activities and skills*, which are often associated with nonverbal mannerisms and emotion. When such mimicry occurs repeatedly, it leads to permanent retention of others’ reactions. One classic example of this is the acquisition of accents. Another is emotional contagion – “catching” of the others’ somatic and feeling states. Importantly, note that individuals’ reactions caused by contagion processes are not typically recognized as being “caused” by the model, but are viewed as one’s own response to the environment. This is important because, given individuals’ ignorance of their external origin, mimicked behaviors and feelings become privatized in the same way as any sensations (e.g., arousal and mood) do, when subject to internal (mis)attribution (Schwarz, 2011). For example, when others laugh at a joke, individuals will too, even though they actually missed the punch line (Provine, 2001). When it is not obvious to individuals that others caused their mirth, contagious laughter may lead them to decide the joke is genuinely funnier (Bush et al., 1989). Similarly, evaluations of a video program converge more when subjects can see their partner, an effect mediated by levels of mimicry (Ramanathan and McGill, 2007).

Critically, humans not only adopt others’ emotional displays, but also others’ postures (Bernieri, 1988). Bodily states can in turn can influence perception, cognition and motivation (for review, see Winkielman et al., 2015). For example, participants made to adopt an upright, as opposed to slumped, posture show greater task persistence (Riskind and Gotay, 1982) and participants told to lean forward while looking at a stimulus show neural indicators of increased desire (Harmon-Jones and Peterson, 2009). In short, postures, even those externally induced, shape people’s own beliefs and motivations. Clearly, we are not claiming that adopted feelings or postures of others are always mis-attributions (i.e., maladaptive errors). We are claiming, however, that individuals can miss that others are the proximate source of their feelings. Consequently, individuals focus on what “caught” states mean to them, causing internalization of others’ feelings – an essential aspect of socialization. Thus, this unawareness is in some sense “rational”, as it produces adaptive social behavior by utilizing relatively simple cues to produce the right internal states.

## Selectivity of Mimicry for Learning From Ingroups

But why is spontaneous mimicry preferentially employed within ingroups? We propose that mimicry entails a potential cost in terms of acquiring maladaptive behaviors and habits. Specifically, whereas the habits of ingroup members have survived in a similar social environment, the habits of outgroup members have not (Donald, 2005). As this transfer of knowledge from one individual to another is implicit, there is a risk of polluting the receiving individuals with bad information that they are not aware of incorporating. Thus, mimicry should be engaged more in the presence of prior cues that attendant feelings constitute “desirable information.” However, this process should be disengaged when prior cues indicate that it will introduce “undesirable information” into the cognitive system. In other words, one can argue that mimicry operates as an implicit “System 1” process (Evans and Stanovich, 2013) that is moderated in an ecologically rational fashion (Gigerenzer et al., 2011).

What factors should rationally (adaptively) modify individuals’ tendency towards mimicry? Humans form groups around similarities in values and priorities, indicating that shared feelings are essential to social function (e.g., McPherson et al., 2001). Humans need to have shared valuations during collective actions of any kind, from deciding what to eat to how to punish a crime. Thus, the characteristics that mark someone as a member of an “ingroup” are much the same as the conditions under which it is adaptive to share feelings (Cikara et al., 2011). Ingroup members’ feelings are highly informative of what our own feelings should be, or would be if we had more experience. An obvious example is a child’s tendency to become afraid in response to a parent’s expression of fear. Thus, greater mimicry should rationally occur when the model is regarded as a good source of information about proper reactions, and ingroup membership serves as a good proxy for these criteria. Of course, as individuals gain experience with complex social environments, they will learn that some characteristics of others correlate with the rewards of spontaneous mimicry. Such cues will include likeability of others, outward markers of ingroup membership, and their status within the ingroup (e.g., “boss”). High-status individuals, as “opinion leaders” will likely be more profitably mimicked by social actors seeking to coordinate feelings with their group. Sometimes individuals will also implicitly learn that certain social situations call for complementary, or counter-mimicry behaviors, such as, for example, smiling submissively when a high status individual is frowning (Carr et al., 2014). The ability to use context to determine whom to mimic and how is crucial, and reflects on our social competence (Kavanagh et al., 2011).

Lastly, note that on our account high-fidelity (detailed) reproduction should be limited to certain contexts. A review of the facial mimicry literature by Hess and Fischer (2014) is consistent with this view, arguing that much of what researchers assumed was mimicry of facial expressions is really about responding to valenced stimuli. For example, smiles are positive stimuli, and like all positive stimuli (e.g., candy) individuals smile in response to them. But this is not mimicry, and in fact in this case individuals do not reproduce the detailed features of the observed expressions, just its affective elements. Some evidence suggests that mimicry involving detailed reproduction of emotional expressions appears more strongly in situations where there is joint attention to the same stimulus between the interactants (Bourgeois and Hess, 2008). On our account, this makes sense if mimicry is part of learning how to feel about something from others. Thus, while repetition of other people’ action and expressions sometimes appears undiscerning, the implicit mimicry process is likely tuned to learning a deeper structure of values, reflecting their preferences, goals, and group affiliations (Carr and Winkielman, 2014).

## Affiliation as a Secondary Outcome of Mimicry: Why Mimics are Liked, But Only If Subtle and Approximate

We propose that mimicry is advantageous to the mimic because it facilitates cognitive and affective convergence with the model. If so, then *as an observable action*, mimicry is an honest signal to the model that (i) the sender views the receiver as a good source from which to learn bodily actions and emotional responses, and (ii) the sender is motivated to converge in attitudes, choices, and values. In other words, not only do new individuals benefit from learning adaptive non-verbal behaviors from ingroup members, but ingroup members also benefit from new individuals honestly learning their non-verbal behaviors. Note also that the chances of a new ingroup member exploiting learned habits for nefarious goals is limited in an ingroup setting, where joint action and cooperation is likely. Thus, if *socio-cultural learning* is the major reason for mimicry, then models should respond positively to mimicry, as it signals the likelihood of useful, predictable, high-quality interactions and joint actions. Thus, mimicry may be the proximate means by which affiliation is achieved, even though we may not be aware of mimicry. Nevertheless, the overall dynamic may be mediated by the contextual variables discussed earlier (e.g., likeability, or ingroup and social status). It is also plausible that broad contextual cues similarly drive strategic or at least goal-dependent mimicry (see Heyes, 2011 for discussion of how contextual variables could moderate spontaneous imitation).

However, if models respond to mimicry positively by affiliating with the mimic, then the anticipation of this response creates incentives to mimic others *in order to gain their affiliation* (potentially for Machiavellian purposes) rather than for acculturation purposes. Therefore, models face the problem of discriminating true vs. false signals of affiliation. One way models can solve this problem is by forming an expectation of the “proper” amount and timing of mimicry. So, if the prior knowledge about a new individual generates an expectation of low levels of mimicry, then high levels of mimicry cause distrust. A related set of cues comes from fidelity, timing and subtlety. High-fidelity mimicry – very precise reproduction and timing – arouses suspicion (unless it occurs in entrainment-like settings, Wiltermuth and Heath, 2009; Manson et al., 2013). The reason is because such mimicry requires strategic cognition, possibility directed at temporary goals, rather than implicit cognition reflecting stable, habitual goals. Note that many non-verbal behaviors function as genuine signals of internal states when produced spontaneously (e.g., Duchenne smile and unbidden laughter), but can also be faked (Boone and Buck, 2003; Bryant and Aktipis, 2014). Just like spontaneous laughter (ibid), spontaneous mimicry is more indicative of a true cooperative disposition than is its deliberate twin. Mimicry behaviors might be especially informative because they often emerge in the course of typical social interaction, even when working memory is loaded (van Leeuwen et al., 2009).

## Mimicry of Inconsequential Actions

Finally, readers might wonder how our account explains why affiliation is produced even by mimicry of seemingly inconsequential gestures (e.g., leg crossing), or actions produced by artificial agents. First, the consequentiality of non-verbal behaviors is generally hard for individuals to assess, partly because they lack explicit insight into these behaviors (Burgoon et al., 2010). Second, arbitrariness of a behavior is sometimes key to its role as a group marker (Legare et al., 2015). Third, as stated, individuals rely on heuristic, global evaluation of the usefulness of engaging in mimicry in general, rather than a comprehensive evaluation of the costs or benefits of a specific mimicry behavior. Consistently, evidence suggests that overall imitative tendencies are moderated by global goal states, but that imitation of specific behaviors is not (Heyes, 2011). Finally, mimicry of even inconsequential behaviors will still signal to models that the mimic considers them as good source of implicit information (which, again, implies the possibility for beneficial interaction).

## Conclusion

Mimicry leads to gradual acquisition of imitated behaviors (gestures, postures, expressions, accents, and mannerisms) and later reproduction of similar behaviors even in the model’s absence. This process is principally developmental, with children learning not only what and who to mimic but also how to mimic, with their skills becoming more implicit and more attuned to ‘non-verbal’ accents of their ingroup. Throughout, children learn expectations for the proper degree and timing, which later allows for discounting of blatant mimicry. Though the importance of some mimicked behaviors is hard for the individuals to establish, we argue that overall this process has symmetrically positive consequences for both individual socialization and group function. This is because mimicry entails coordinated affect, actions or reactions – thus signaling to the model that the mimic is likely to have, or to develop, the shared values necessary for sociality. Because mimicry is a valid signal that the mimic is likely to have beneficial social interactions with the model and its group, affiliation with the mimic is also adaptive for the group. The positive reaction and use of this signal is, just like a smile, complicated by incentives for Machiavellian exploitation. However, the risk of such exploitation is reduced by the benefits of selectively incorporating ingroup values as one’s own, the costs of internalizing maladaptive behaviors and feelings of the outgroup, and by the presence of spontaneity cues in timing and fidelity.

## Author Contributions

The authors, LK and PW cooperated on developing the theoretical framework and preparing the manuscript.

## Conflict of Interest Statement

The authors declare that the research was conducted in the absence of any commercial or financial relationships that could be construed as a potential conflict of interest.
